# Human Chorionic Gonadotropin-Mediated Induction of Breast Cancer Cell Proliferation and Differentiation

**DOI:** 10.3390/cells10020264

**Published:** 2021-01-29

**Authors:** Ilaria Dando, Cristian Andres Carmona-Carmona, Nicola Zampieri

**Affiliations:** 1 Department of Neurosciences, Biomedicine and Movement Sciences, University of Verona, 37134 Verona, Italy; cristianandres.carmonacarmona@univr.it; 2 Paediatric Fertility Lab, Department of Surgery, Dentistry, Paediatrics and Gynaecology, Woman and Child Hospital, Division of Pediatric Surgery, University of Verona, 37134 Verona, Italy; nicola.zampieri@aovr.veneto.it

**Keywords:** human chorionic gonadotropin (hCG), breast cancer cells, MCF7, cancer stem cells, cancer proliferation

## Abstract

Human chorionic gonadotropin (hCG) is a hormone that specifically binds to luteinizing hormone receptor (LHR) and exerts several roles, including the support of pregnancy and fetal gonadal steroidogenesis. Since hCG is also expressed by some tumor types, like breast cancer, many efforts have been made to study its role in neoplesia, with some studies showing a cancer-supportive role and others showing a cancer-protective role. A critical examination of the literature highlighted that the in vitro effect of hCG has been tested in the presence of fetal serum, which contains other gonadotropins, in the culture medium. Thus, we hypothesized that the use of serum in the cell culture medium might influence the cell response to the hCG treatment due to the presence of other hormones. Thus, we analyzed the in vitro effect of highly purified hCG on cell proliferation and the activation of the down-stream signal transduction pathway in three breast cancer cell lines, particularly focusing on MCF7, cultured in serum-deprived conditions. Our data show that hCG increases cell proliferation and activates the down-stream target Akt, together with a decrease of the LHR mRNA expression level. Finally, we also tested the differentiation capacity of hCG on MCF7 cancer stem cells (CSCs) and show that it favors the proliferation and differentiation of these cells, thus suggesting that hCG also renders cells more able to colonize and invade the organs.

## 1. Introduction

Human chorionic gonadotropin (hCG) is a glycoprotein hormone that belongs to the gonadotropin hormone family, which also includes luteinizing hormone (LH) and follicle-stimulating hormone (FSH). hCG is a heterodimeric protein with two subunits: the alpha-subunit that is identical to that of LH, FSH, and thyroid-stimulating hormone (TSH), and the beta-subunit that is specific to hCG alone. These hormones bind to specific G protein-coupled receptors (GPCRs): LH and hCG bind to luteinizing hormone receptor (LHR), whereas FSH binds to follicle-stimulating hormone receptor (FSHR). LHR is principally expressed in the ovary and testicular cells, and its activation is fundamental to activating the hormone functions. hCG is mainly produced by the placenta during pregnancy, but it is also expressed at lower levels in other tissues, including the testis, prostate, thymus, skeletal muscles, and pituitary gland, by exerting an autoregulatory growth-promoting role [1]. Its role in normal tissue seems to be mainly autoregulatory by favoring the stimulation of cell growth [2]. Despite the molecular effect of hCG on fertility being a clinical subject matter [3,4], its role in cancer development has not yet been completely assessed and remains controversial, with some authors showing that hCG exerts anti-tumoral effects by decreasing cell proliferation [5], and others showing that it acts as a pro-cancer hormone [6,7]. In support of the protective role of hCG against breast cancer, in vitro studies demonstrated that hCG decreased cancer cell proliferation [8], and in vivo models showed that hCG administration reduced the risk of carcinogen-induced breast cancer in rats, together with the induction of apoptosis [9,10]. Instead, concerning the pro-tumorigenic role of hCG, it has been shown that some tumors, including breast, bladder, and testicular cancer [11,12], express this hormone at high levels, thus making it a potential tumor marker and a tool to monitor disease progression [13]. An interesting study performed on breast cancer patients showed that β-hCG mRNA was present in the blood of 80% of primary breast cancers, whereas it was not expressed in negative controls, representing a potential marker for the detection of metastatic breast cancer cells. Furthermore, transgenic mice overexpressing β-hCG have been shown to develop multiple neoplasms, including breast cancer, also showing that hCG can up-regulate Wnt7b and Wnt5b, thus contributing to pregnancy-induced breast cancer in humans [14]. In vitro studies also showed that β-hCG promotes cancer cell invasion in different types of tumors [15,16].

Another important element linked to the action of hCG is the expression level of LHR, whose role as a pro- or anti-tumor agent is controversial as well. Indeed, LHR expression has been reported to be associated with favorable tumor characteristics [17] and, controversially, in another study it was reported to be increased in invasive breast cancer cases in comparison to preinvasive specimens.

Since the effect and the role of hCG in cancer cells still remain unclear, we critically examined different studies in which hCG has been tested in vitro and hypothesized that a general common issue is the presence in the culture medium of fetal serum, the formulation of which contains both LH and FSH. Among all the components of fetal serum, we suggest that the presence of these two hormones might activate the same signal transduction pathway as hCG, rendering it impossible to discern the real effect of hCG on the tested cells. For this reason, the aim of this study was to test the effect of highly purified hCG in breast cancer cell lines, particularly focusing on MCF7 cells, cultured without serum. Furthermore, since Liao et al. showed that hCG stimulates cells to differentiate [8], we also analyzed, in serum-deprived conditions, the effect of hCG on MCF7 cancer stem cell (CSC) proliferation and differentiation.

## 2. Materials and Methods

This prospective observational study was approved by the Pediatric Fertility Lab internal review board (09/2020).

### 2.1. Drugs and Chemicals

Highly purified human chorionic gonadotropin (hCG) extracted from pregnant women’s urine was kindly provided by IBSA Italia (Lodi, Italy).

### 2.2. Cell Lines

To investigate the effect and the role of hCG in cancer cells, the following cell lines were used: a pancreatic ductal adenocarcinoma cell line (PaCa44) and breast cancer cell lines (MCF7, T47D, and MDA-MB-231). Due to its origin, the PaCa44 cell line does not express LHR; thus, it was identified as control cell line. PaCa44 cells were grown in RPMI-1640, whereas MCF7, T47D, and MDA-MB-231 cells were grown in DMEM-Glutamax, both supplemented with 10% fetal bovine serum (FBS) (generally containing FSH and LH at different concentrations depending on the lot, as reported by different manufacturers’ sheets) and 50 µg/mL gentamicin sulfate (all from Thermo Fisher Scientific, Waltham, MA, USA), and maintained at 37 °C with 5% CO_2_. The PaCa44 cell line was kindly provided by Dr. Aldo Scarpa (University of Verona, Verona, Italy) and the MCF7, T47D, and MDA-MB-231 cell lines were purchased from ATCC (Manassas, VA, USA).

Cancer stem cells (CSCs) were obtained as previously described by us and other research groups [18,19,20]. Briefly, adherent MCF7 cells were washed twice in 1× PBS (Thermo Fisher Scientific, Waltham, MA, USA) and then cultured in stem-specific medium, i.e., DMEM/F-12 without glucose (US Biological Life Sciences, Salem, MA, USA) supplemented with 1 g/L glucose, B27, 1 µg/mL fungizone, 1% penicillin/streptomycin (all from Gibco/Life Technologies, USA), 5 µg/mL heparin (Sigma/Merck, St. Louis, MO, USA), 20 ng/mL epidermal growth factor (EGF), and 20 ng/mL fibroblast growth factor (FGF) (both from PeproTech, London, UK) and cultured in ultra-low-attachment flasks (Sarstedt, Nümbrecht, Germany).

CD44^+^/CD24^−^ CSCs were sorted using Becton Dickinson FACSAria Fusion (BD Biosciences, San Jose, CA, USA). Briefly, to sort tumor cells with CD44-positive and CD24-negative (CD44^+^/CD24^−^) marker expression, MCF7 cells were washed with phosphate-buffered saline (PBS) and harvested with 0.05% trypsin/0.025% EDTA. Cells were then subjected to antibody binding, i.e., combinations of fluorochrome-conjugated monoclonal antibodies against human CD44 (APC) and CD24 (PE) or their respective isotype controls at the concentration recommended by the manufacturer, and incubated at room temperature in the dark for 20 min. After that, cells were washed twice with PBS + 1 mM EDTA + P/S + 1% FBS and resuspended in the same buffer at a concentration of 20–30 × 10^6^ cells/mL of buffer. Anti-human CD44 (APC) and relative isotype were obtained from BioLegend (San Diego, CA, USA), whereas anti-human CD24 (PE) and relative isotype were obtained from MACS. Sorted cells were then cultured in the stem-specific medium, as reported above, for at least one week.

Before hCG treatment, adherent cells were maintained for 16 h in an FBS-free medium; then, cells were treated with the indicated doses and for the indicated time with hCG by keeping cells in the medium without FBS.

Bright field cell images were acquired using an inverted microscope (Axio Vert. A1, Zeiss, Oberkochen, Germany), and the diameters of more than 80 spheres for each set of conditions of each biological replicate were measured using ImageJ software (ImageJ, http://rsb.info.nih.gov/ij/, 1997–2008).

### 2.3. Immunofluorescence Analysis of LHR-Positive Cells

PaCa44 and MCF7 cells were fixed in 4% paraformaldehyde for 8 min and washed 3 times with PBS for 5 min each. To saturate unspecific binding sites, the cells were incubated for 45 min at RT with a blocking solution containing 5% BSA in PBS. Samples were then incubated overnight at 4 °C with anti-LHR (1:200; Thermo Fisher #8G9A2, Waltham, MA, USA) primary antibody diluted in blocking solution. After 3 washes with PBS of 10 min each, cells were incubated for 1 h at RT in the dark with specific secondary antibody (1 µg/mL) conjugated with Alexa Fluor-488 (Molecular Probes, Eugene, OR, USA). Samples were mounted in anti-bleaching medium (Dako Fluorescent Mounting Medium, Agilent Technologies, Santa Clara, CA, USA) and examined by fluorescence microscope (Axio Vert. A1, Zeiss).

### 2.4. Immunoblot Analysis

Immunoblot assays were performed, as previously described [21], by analyzing whole-cell lysate. Membranes were blocked in 5% low-fat milk in TBST (50 mM Tris pH 7.5, 0.9% NaCl, 0.1% Tween 20) for 1 h at room temperature (RT) and probed overnight at 4 °C with anti-phospho(Ser473)-Akt (1:2000; Cell Signaling #4060, Danvers, MA, USA) and anti-Akt (1:1000; Cell Signaling #9272). Horseradish-peroxidase-conjugated anti-mouse (1:10,000; KPL #074-1806) or anti-rabbit IgGs (1:2000; Cell Signaling #7074) were used as secondary antibodies. The immunocomplexes were visualized by chemiluminescence using the ChemidocMP imaging system (Bio-Rad Laboratories, Hercules, CA, USA), and the intensity of the chemiluminescence response was measured by processing the image using NIH Image J software (http://rsb.info.nih.gov/nih-image/). Ponceau S staining was used to confirm loading in different lanes.

### 2.5. RNA Extraction and qPCR

RNA extraction and real-time quantitative PCR (qPCR) were performed as previously described [21]. The primers used were LHR forward, 5′-GCTGCGATTAAGACATGCCA-3′, LHR reverse, 5′-AGAAGGCCACCACATTGAGA-3′; FSHR forward, 5′-GGCCATGCTCATCTTCACTG-3′, FSHR reverse, 5′-ATAGAGGAAGGGGTTGGCAC-3′; Androgen Receptor (AR) forward, 5′-CCCACTTGTGTCAAAAGCGA-3′, AR reverse, 5′-GCAGCTTCCACATGTGAGAG-3′; Zeb-1 forward, 5′-GTTACCAGGGAGGAGCAGTGAAA-3′, Zeb-1 reverse, 5′-GACAGCAGTGTCTTGTTGTTGTAGAAA-3′; OCT3/4 forward 5′-GACAGGGGGAGGGGAGGAGCTAGG-3′, OCT3/4 reverse 5′-CTTCCCTCCAACCAGTTGCCCCAAAC-3′; Nanog forward 5′-AGTCCCAAAGGCAAACAACCCACTTC-3′, Nanog reverse 5′-TGCTGGAGGCTGAGGTATTTCTGTCTC-3′; SOX2 forward 5′-GGGAAATGGGAGGGGTGCAAAAGAGG-3′, SOX2 reverse 5′-TTGCGTGAGTGTGGATGGGATTGGTG-3′; CDH1 forward 5′-GACACCAACGATAATCCTCCGA-3′, CDH1 reverse 5′-GGCACCTGACCCTTGTACGT-3′; RPLP0 forward 5′-ACATGTTGCTGGCCAATAAGGT-3′, and RPLP0 reverse 5’-CCTAAAGCCTGGAAAAAGGAGG-3′.

### 2.6. Cell Proliferation Assay

MCF7, T47D, and MDA-MB-231 cells and CSCs were plated in 96-well cell culture plates (5 × 10^3^ cells/well) and incubated at 37 °C with 5% CO_2_. For the MCF7, T47D, and MDA-MB-231 cells, cell viability was measured by crystal violet assay (Merck Millipore, Burlington, MA, USA) according to the manufacturer’s protocol, and absorbance was measured by spectrophotometric analysis (A595nm). The crystal violet assay is designed to work with adherent cells, and when cells die, they detach from the surface of the plate. Thus, this assay is suitable for in vitro cell proliferation and cell cytotoxicity studies, as reported in several manufacturer’s sheets. For MCF7 CSCs, viable cells were counted by trypan blue dye exclusion.

### 2.7. Statistical Analysis

ANOVA (post hoc Bonferroni) analysis via GraphPad Prism 5 (GraphPad Software, Inc., San Diego, CA, USA) or Student’s *t*-test (two-tailed) was conducted. *p*-values of <0.05, <0.01, or <0.001 were considered to indicate significant difference (for details, see figure legends).

## 3. Results and Discussion

### 3.1. hCG Stimulates Breast Cancer Cell Proliferation

**Figure 1:**
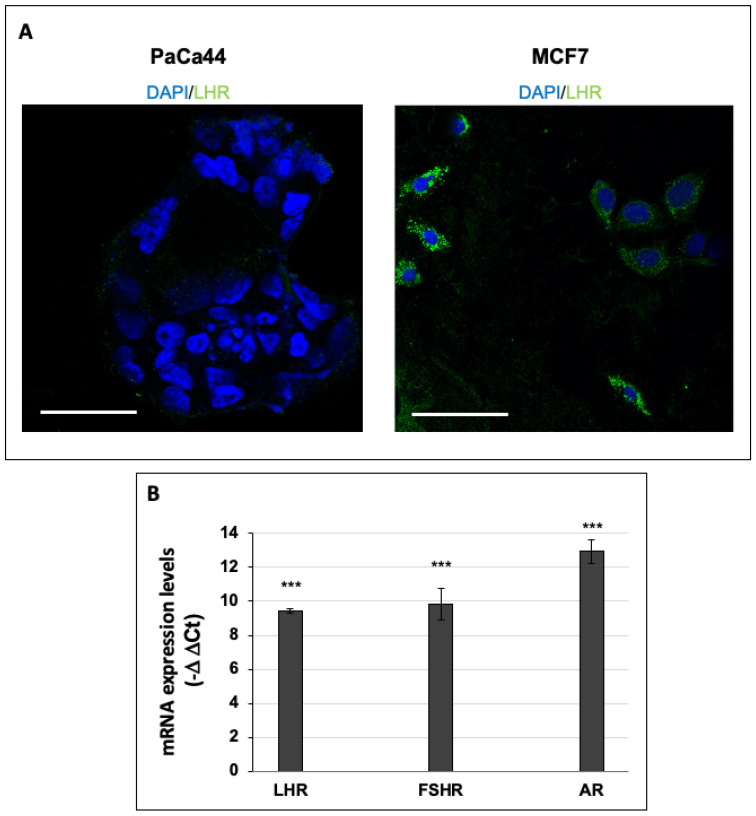
(**A**) Representative immunofluorescent images of luteinizing hormone receptor (LHR) expression in PaCa44 and MCF7 cells. Scale bar: 50 µm. (**B**) mRNA expression levels of LHR, follicle-stimulating hormone receptor (FSHR), and AR expressed as “–delta delta Ct” values in relation to the housekeeping gene expression levels (RpLp0) in MCF7 cells relative to the pancreatic ductal adenocarcinoma cell line PaCa44, used as a control. Values are the means (±SD) of three independent biological replicates. Statistical legend: *p* < 0.001 (***) for MCF7 cells relative to PaCa44 cells.

In order to study the effect of human chorionic gonadotropin (hCG) on breast cancer cells, we firstly analyzed the expression of the hCG-specific receptor LHR through confocal microscopy in MCF7 cells in comparison to the control cell line PaCa44, which does not express LHR due to its origin, i.e., pancreatic ductal adenocarcinoma. Indeed, the immunofluorescent analysis showed that LHR was not expressed by PaCa44, whereas MCF7 cells expressed the receptor at high levels in all the cells with a wide presence of fluorescent spots all over the cell surfaces (Figure 1A). Furthermore, in addition to the analysis of LHR protein expression, we also analyzed the mRNA expression patterns of LHR and two other hormone receptors, FSHR and AR, which emerged as useful markers for the refinement of breast cancer subtype classification [22]. We extended the analysis of mRNA receptor levels to two other breast cancer cell lines, i.e., T47D and MDA-MB-231. Our data show that MCF7, T47D, and MDA-MB-231 cells expressed the mRNA of LHR, FSHR, and AR at high levels in comparison to control cells (PaCa44 cells) (Figure 1B and Figure S1), thus supporting the use of these cells, particularly MCF7, to perform studies on the effect of hCG.

**Figure 2:**
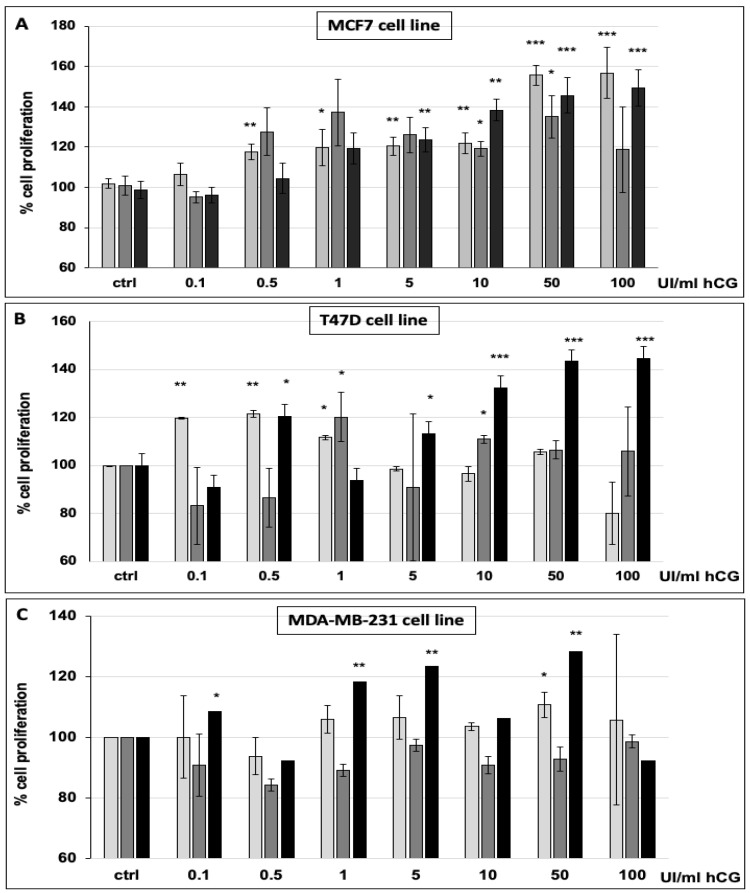
Cell proliferation of MCF7 (**A**), T47D (**B**), and MDA-MB-231 (**C**) cultured in FBS-deprived medium and treated with 0.1, 0.5, 1, 5, 10, 50, or 100 UI/mL human chorionic gonadotropin (hCG) for 24, 48, or 72 h. Histogram legend: 24 h treatment (light gray); 48 h treatment (dark gray); 72 h treatment (black). Values are the means (±SD) of three independent biological replicates. Statistical legend: *p* < 0.5 (*), *p* < 0.01 (**), *p* < 0.001 (***) for treated cells relative to untreated cells (control).

Based on the observation that fetal bovine serum (FBS), generally used to culture in vitro cells, contains the hormones LH and FSH (as reported in Section 2), we analyzed the proliferation of the three breast cancer cell lines cultured and treated with hCG in FBS-rich and FBS-deprived media. As shown in Figure S2, MCF7, T47D, and MDA-MB-231 cells cultured and treated for 72 h in an FBS-complete medium with increasing doses of hCG did not change in terms of their proliferation in comparison to untreated cells grown in the same culture condition. This analysis was also performed for shorter hCG treatment periods of 24 and 48 h, showing the same effect (data not shown). MCF7, T47D, and MDA-MB-231 cells cultured in an FBS-deprived medium and treated with increasing doses of hCG for 24, 48, and 72 h showed a significant increment of proliferation (Figure 2A–C). In detail, we observed that at a concentration of 10 UI/mL, hCG significantly stimulated MCF7 cells to proliferate at all three time points, with an increased extent at higher doses. Furthermore, a 60% increase in proliferation occurred in response to both 50 and 100 UI/mL of hCG at 24 and 72 h (Figure 2A). The proliferation of the T47D and MDA-MB-231 cells also significantly increased after hCG treatment, with a stronger extent at 72 h (Figure 2B,C). Our data show that, in serum-free conditions, breast cancer cells are stimulated by hCG to proliferate. Hence, the elevated expression of LHR receptors in these cells supports the hypothesis that hCG favors cancer cell growth. These in vitro data indicate that the composition of the medium is critical to evaluating the effect of hCG, and that it is recommendable to use a serum-free medium to evaluate the hormonal effect.

### 3.2. hCG Increases the Phosphorylation of Akt and Regulates the Expression of Hormone Receptors

**Figure 3:**
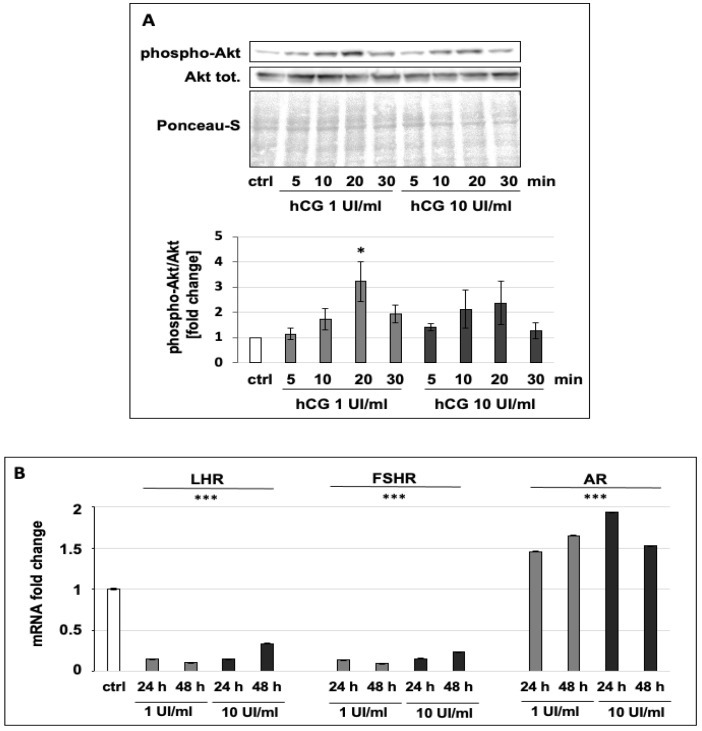
(**A**) Representative Western blots of phospho- and total-Akt normalized on Ponceau S. The densitometric analysis is the average of three biological replicates. (**B**) mRNA expression levels of LHR, FSHR, and AR relative to the housekeeping gene expression levels (RpLp0) in MCF7 cells untreated or treated with 1 or 10 UI/mL hCG for 24 or 48 h in a serum-free medium. Values are the means (±SD) of three independent biological replicates. Statistical legend: *p* < 0.5 (*) and *p* < 0.001 (***) for treated cells relative to untreated MCF7 cells (control).

To confirm that hCG stimulates the LHR down-stream pathway, we analyzed the phosphorylation level of the kinase Akt, which has been described to be phosphorylated and then activated after LHR stimulation [23]. In order to analyze modulation of the LHR down-stream pathway, we chose two hCG concentrations, 1 and 10 UI/mL, at which MCF7 cell proliferation was moderately induced (Figure 2B). Indeed, the immunoblot analysis showed that both 1 and 10 UI/mL hCG increased the level of the phosphorylated form of Akt after 20 min of stimulation (Figure 3A). Interestingly, the Akt phosphorylation was strongly decreased after 30 min, suggesting that the effect of the hormone takes place within a short range of time. Another evaluation of the effect of hCG was made via analysis of LHR mRNA levels, the expression of which has been described to be decreased after LHR stimulation. Indeed, it was previously demonstrated that the activation of LHR brings about a decrease in its mRNA expression due to the induction of miRNA-122 [24]. In line with this, our data show that the LHR mRNA level was significantly decreased after hCG treatments at both 1 and 10 UI/mL for 24 and 48 h (Figure 3B). Furthermore, we also analyzed the mRNA levels of FSHR and AR and observed that the FSHR mRNA expression level was decreased, whereas that of AR was increased (Figure 3B), thus opening the way to further hypotheses about the pleiotropic effect of hCG in breast cancer cells.

### 3.3. hCG Stimulates Breast Cancer Stem Cell Proliferation and Differentiation

**Figure 4:**
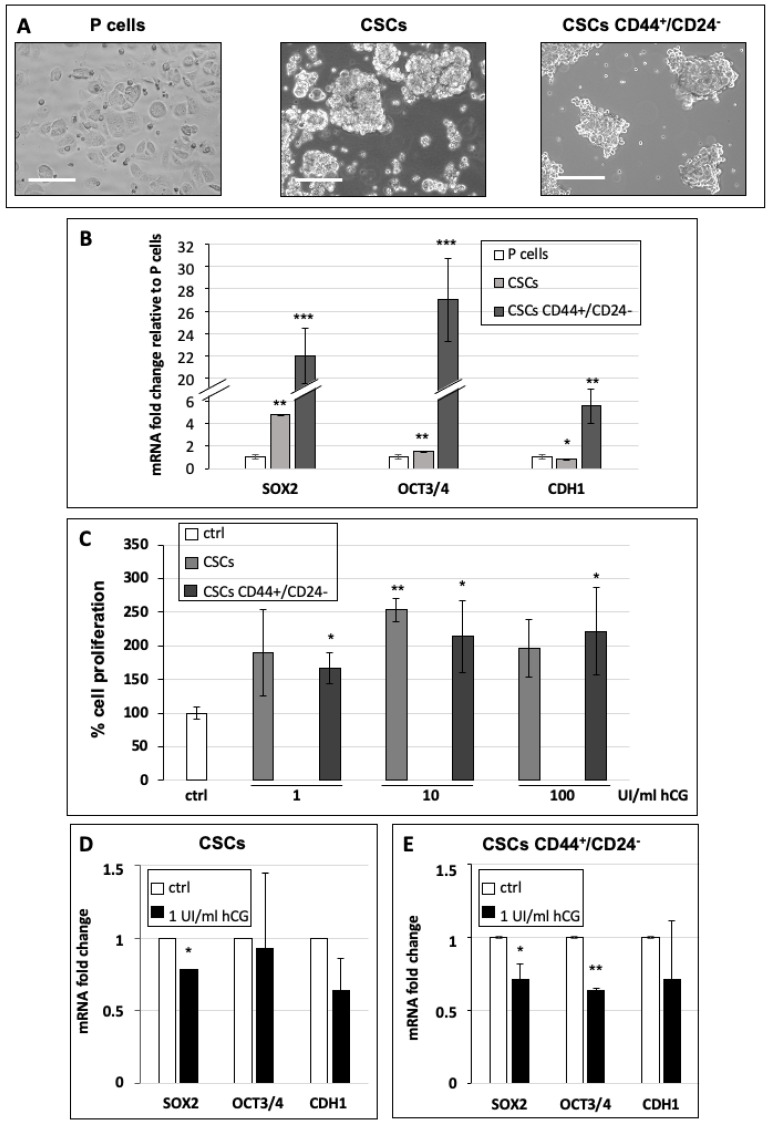
(**A**) Representative images of MCF7 parental (P) cells, MCF7 cancer stem cells (CSCs), and CSCs expressing CD44^+^/CD24^−^. Scale bar: 50 µm. (**B**) mRNA expression levels of SOX2, OCT3/4, and CDH1 relative to the housekeeping gene expression levels (RpLp0) in MCF7 P cells (white bar), CSCs (light gray bar), and CD44^+^/CD24^−^ CSCs (dark grey bar). (**C**) Analysis of viable CSCs after treatment with 1, 10, or 100 UI/mL hCG for 72 h. Control cells (white bar), CSCs (light gray bar), and CD44^+^/CD24^−^ CSCs (dark grey bar). mRNA expression levels of SOX2, OCT3/4, and CDH1 relative to the housekeeping gene expression levels (RpLp0) in MCF7 CSCs (**D**) and CD44^+^/CD24^−^ CSCs (**E**) untreated (white bar) or treated with 1 UI/mL hCG (black bar). All the experiments were performed in serum-free medium. Values are the means (±SD) of three independent biological replicates. Statistical legend: *p* < 0.5 (*), *p* < 0.01 (**), *p* < 0.001 (***) for treated cells relative to untreated cells (control).

Since it has been described that hCG favors the differentiation of mammary gland tumor cells [8], we studied the effect of hCG on cancer stem cells (CSCs) derived from the MCF7 cell line (parental cells). In addition, we also tested hCG’s effect on a subclass of CSCs with CD44^+^/CD24^−^ marker expression, which have been shown to have the highest tumorigenic potential in tumor cell invasion and nude mouse xenograft assays [25]. Firstly, in order to confirm the stem properties of the obtained cells, we characterized their capacity to form mammospheres and to express stem and epithelial-to-mesenchymal transition (EMT) markers. Our data show that cells cultured in CSC-specific medium were able to form floating round-shaped aggregates (Figure 4A), representing a stem hallmark [18]. Furthermore, both CSCs and CD44^+^/CD24^−^ CSCs significantly expressed the stem markers SOX2 and OCT3/4 mRNA at high levels. Interestingly, only CSCs expressed low levels of the EMT marker CDH1 (this transcribes for E-cadherin, which is down-regulated in mesenchymal cells) (Figure 4B), in comparison to differentiated MCF7 parental cells, whereas CD44^+^/CD24^−^ CSCs expressed high levels of CDH1, opening the way to future analyses of their propensity to perform EMT. In order to analyze the effect of hCG on CSC proliferation, we treated MCF7 CSCs and CD44^+^/CD24^−^ CSCs with three doses of hCG—1, 10, and 100 UI/mL—for 72 h and evaluated cell growth. In accordance with the data obtained on parental cells (Figure 2), hCG stimulated CSCs and CD44^+^/CD24^−^ CSCs to proliferate, with increased extent at 10 and 100 UI/mL (Figure 4C), further supporting a pro-tumor effect of hCG treatment in this known highly aggressive subclass of cancer cells.

Since it has been shown that CSCs may differentiate under specific stimuli in order to colonize a secondary organ to generate metastases, we also investigated the effect of hCG on the differentiation of CSCs. To study CSC differentiation, we analyzed whether CSC sphere size was decreased by hCG treatment, representing a first hallmark of the loss of stem features. Indeed, there was a strong reduction in sphere size after 100 UI/mL hCG treatment (Figure S3A), and the sphere area was significantly decreased after treatments with 1, 10, and 100 UI/mL hCG (Figure S3B). Interestingly, at higher doses of hCG, the cell sphere size progressively decreased, suggesting a stronger differentiation capacity of hCG at higher doses. It is noteworthy that cell viability was maintained at a high level (more than 85%) in all the conditions (for details, see Section 2). To confirm these data at the mRNA level, we analyzed the stem and EMT marker expression in CSCs and CD44^+^/CD24^−^ CSCs treated with 1 UI/mL of hCG for 24 h (Figure 4D,E). Our data indicate that CSCs treated with hCG showed significantly decreased expression of SOX2, by 0.78 fold, in comparison to untreated cells, whereas OCT3/4 and CDH1 mRNA levels were unchanged (Figure 4D); this suggests that in these cells, SOX2 may be one of the first actors in the differentiation process. In CD44^+^/CD24^−^ CSCs, hCG decreased the expression of both SOX2 and OCT3/4 to a significant extent (Figure 4E).

## 4. Conclusions

Because different studies that investigated the effect of hCG in cancer cells, including the breast cancer cell line MCF7, have reported controversial results [26], we hypothesized that the presence of serum, which generally contains LH and FSH, in the culture medium may influence cell response to the exogenous administration of hCG. Thus, to shed light on the effect of this hormone in cancer cells, we analyzed the effect and the role of hCG treatment on breast cancer cells in serum-free conditions. Overall, our data showed that hCG stimulates cell proliferation, allowing us to hypothesize that the propensity of breast cancer cells to express the hormone receptor LHR renders them highly sensitive to hCG treatment by increasing their proliferative capacity. In addition, we also showed that hCG is able to increase the differentiation of cancer stem cells, thus potentially rendering them more able to colonize and invade the primary organ or to metastasize. Thus, our data suggest that, in order to study the effect of hCG in vitro, it is desirable to culture cells in the absence of serum in order to avoid interference by the other hormones contained in it. From a clinical point of view, further studies of the role of hCG in breast cancer may be particularly important in pregnancy-associated breast cancers. Finally, this study also offers new perspectives for future research about the effect of hCG, not only in cancer cells and CSCs, but also on human cells and human stem cells, focusing on infertility and fertility preservation.

## Supplementary Materials

The following are available online at https://www.mdpi.com/2073-4409/10/2/264/s1, Figure S1: mRNA expression levels of LHR, FSHR, and AR expressed as “–delta delta Ct” values in relation to the housekeeping gene expression levels (RpLp0) in T47D and MDA-MB-231 cells relative to the pancreatic ductal adenocarcinoma cell line PaCa44, used as a control. Values are the means (±SD) of three independent biological replicates. Statistical legend: *p* < 0.001 (***) for T47D or MDA-MB-231 cells relative to PaCa44 cells, Figure S2: Cell proliferation of MCF7 (A), T47D (B), and MDA-MB-231 (C) cultured in FBS-complete medium and treated with 0.1, 0.5, 1, 5, 10, 50, or 100 UI/mL hCG for 72 h, Figure S3: Representative images (A) and quantification of the sphere area of MCF7 CSCs untreated (control) or treated with 100 UI/mL hCG for 48 h (B). Scale bar: 50 mm. Values are the means (±SD) of three independent biological replicates. Statistical legend: *p* < 0.5 (*), *p* < 0.01 (**), *p* < 0.001 (***) for treated CSCs relative to untreated MCF7 CSCs (control).
