# Supplementary material for: Human Chorionic Gonadotropin-Mediated Induction of Breast Cancer Cell Proliferation and Differentiation
Source: Cells. 2021 Jan 29;10(2):264. doi: 10.3390/cells10020264 (PMC7911704; doi:10.3390/cells10020264)
Supplement: Supplementary file 1 [file cells-10-00264-s001.pdf]

Supplementary:

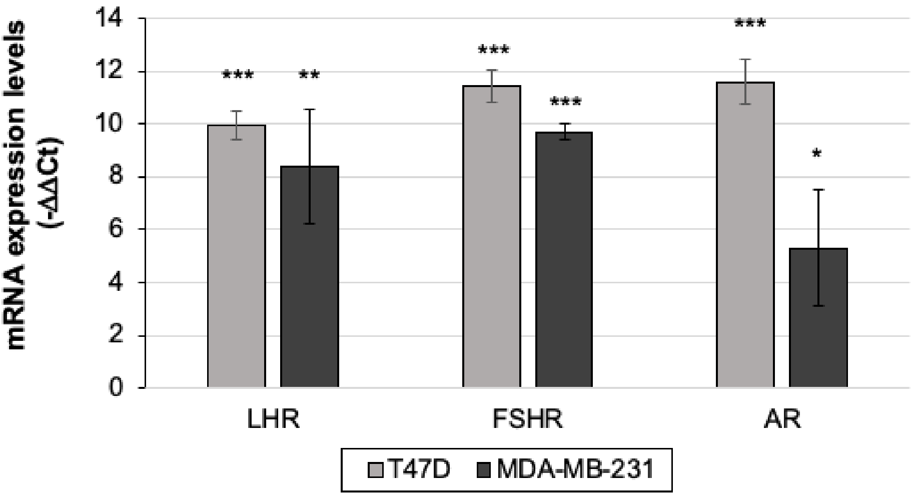

Supplementary Figure 1: mRNA expression levels of LHR, FSHR, and AR expressed as "-delta delta Ct" in relation to the housekeeping gene expression levels (RpLp0) in T47D and MDA-MB-231 cells relative to the pancreatic ductal adenocarcinoma cell line PaCa44, used as control. Values are the means (± SD) of three independent biological replicates. Statistical legend: $p<0.001$ (***)

T47D or MDA-MB-231 cells relatively to PaCa44 cells.

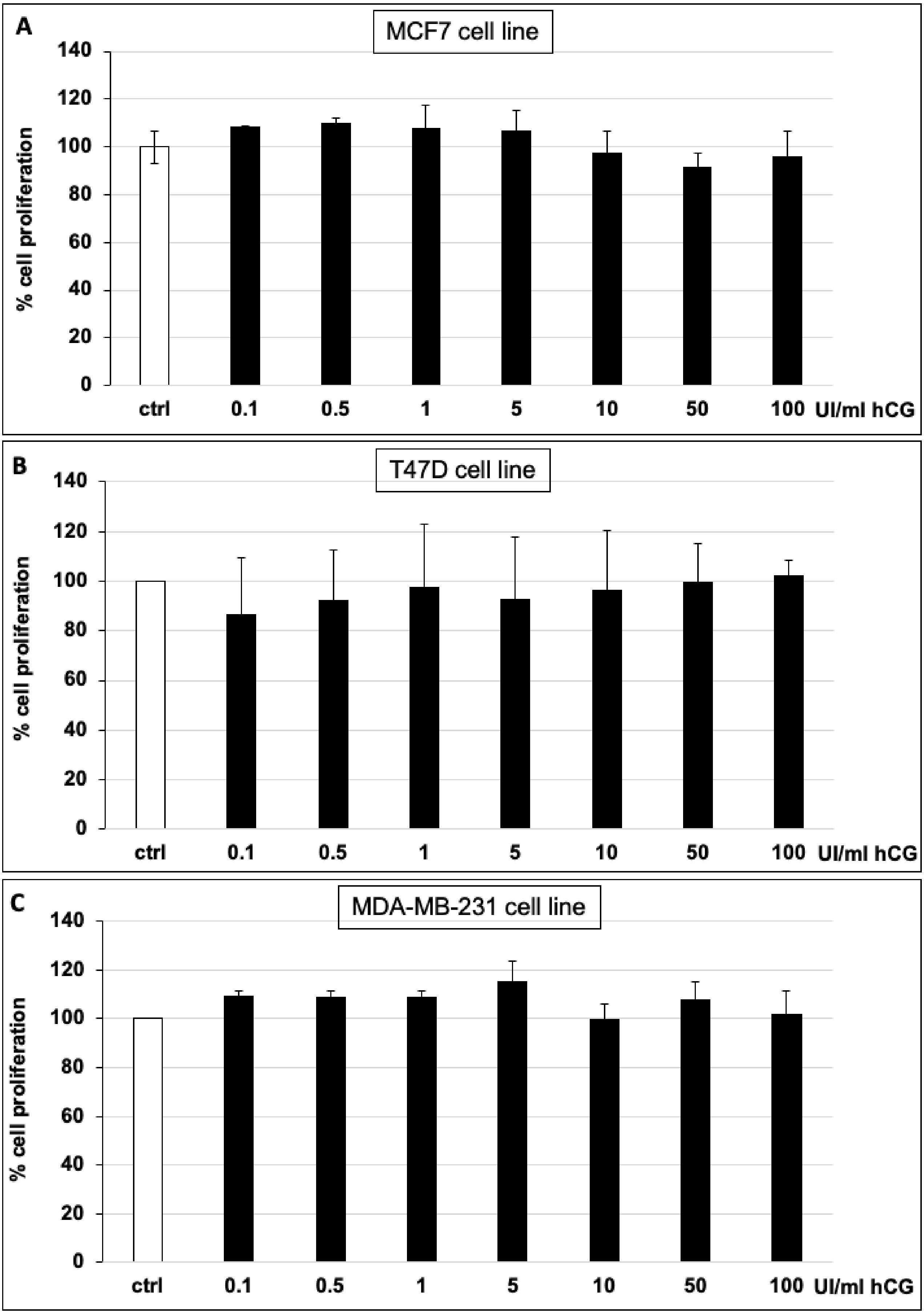

Supplementary Figure 2

Supplementary Figure 2: Cell proliferation of MCF7 (A), T47D (B), and MDA-MB-231 (C) cultured in FBS-complete medium and treated with 0.1, 0.5, 1, 5, 10, 50, and 100 UI/ml hCG for 72 hours.

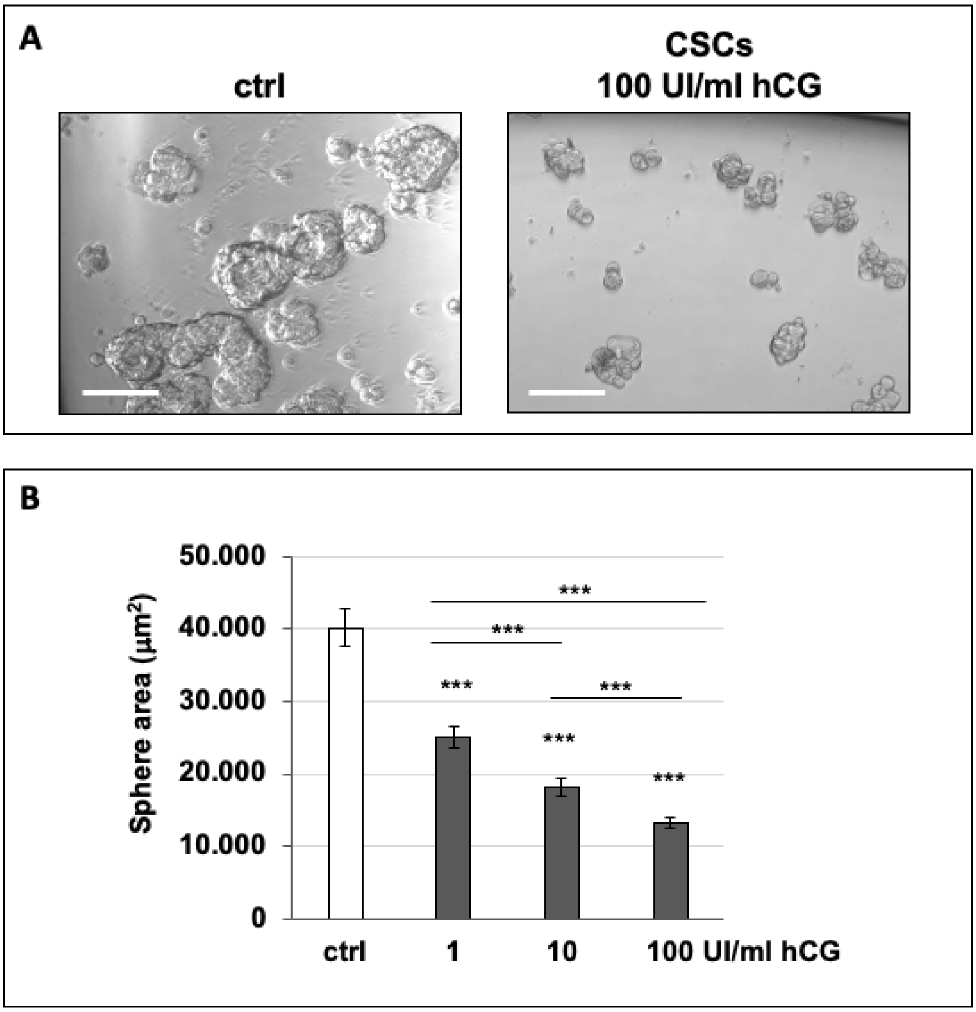

Supplementary Figure 3

Supplementary Figure 3: Representative images (A) and quantification of the sphere area of untreated MCF7 CSCs (control) and treated with 100 UI/ml hCG for 48 h (B). Scale bar: 50 µm. Values are the means (± SD) of three independent biological replicates. Statistical legend: $p<0.5$ (*), $p<0.01$ (**), $p<0.001$ (***) of treated CSCs relative to untreated MCF7 CSCs (control).
